# Longitudinal Effects of 1-Year Smoking Cessation on Human Bronchial Epithelial Transcriptome

**DOI:** 10.1016/j.chest.2022.12.050

**Published:** 2023-01-28

**Authors:** Senani N.H. Rathnayake, Benedikt Ditz, Brigitte W.M. Willemse, National Heart, Lung, and Blood Institute LungMAP Consortium, Hananeh Aliee, Fabian J. Theis, M.C. Nawijn, Wim Timens, Wierd Kooistra, Irene H. Heijink, Brian G.G. Oliver, Maarten van den Berge, Alen Faiz

**Affiliations:** a Respiratory Bioinformatics and Molecular Biology Group, The University of Technology Sydney, Sydney, NSW, Australia; b School of Life Sciences, The University of Technology Sydney, Ultimo, NSW, Australia; c Respiratory Cellular and Molecular Biology Group, Woolcock Institute of Medical Research, Sydney, NSW, Australia; d Groningen Research Institute for Asthma and COPD, University of Groningen, Groningen, The Netherlands; e Department of Pulmonary Diseases,University Medical Centre Groningen, Groningen, The Netherlands; f Department of Paediatrics and Pulmonology, Beatrix Children's Hospital, University Medical Centre Groningen, Groningen, The Netherlands; g Department of Pathology & Medical Biology, University Medical Centre Groningen, University of Groningen, Groningen, The Netherlands

To the Editor:

Smoking is known for its adverse health effects, which include increased risk for various pulmonary diseases, such as lung cancer and COPD.^1^ Approximately 80% of patients who experience COPD smoke.^2^ Smoking cessation is the most successful intervention to improve respiratory symptoms in people with COPD who smoke and those without COPD who smoke.^2^ Cross-sectional studies have been done to describe the transcriptome changes that are associated with smoking.^3^ A longitudinal study by Willemse et al^2^ found a reduction in mast cell populations in asymptomatic participants who previously smoked, after 1 year of smoking cessation. In contrast, specific inflammatory cell populations remained the same or increased in participants with COPD who smoke. The current letter is based on transcriptome-wide data in bronchial biopsies from the same study cohort. We first assessed the effects of smoking cessation on the bronchial transcriptome in asymptomatic participants who smoke vs participants with COPD who smoke, and no significant association was found. Thus, the present study investigated the transcriptomic changes in bronchial biopsies of participants who smoked, before and after 1 year of smoking cessation, regardless of disease status, to investigate the overall effect of smoking cessation.

## Patients and Methods

The initial study consisted of 63 subjects with and without COPD, of which 33 successfully quit smoking for 1 year. The 11 patients with COPD and five asymptomatic participants who smoked, who successfully completed the 1-year smoking cessation program, and had bronchial biopsies available with good quality RNA (before and after 1-year smoking cessation) data were used for the current analysis.^2^ The Groningen University Medical Centre’s local medical ethics committee approved the study; all participants gave their written informed consent. Bronchial biopsy specimens were collected as described previously^2^; RNA was extracted with the use of the all prep kit from Qiagen according to the manufacturer’s instructions, and bulk RNA-sequencing was performed with the use of Illumina NovaSeq6000 (Illumina Inc) paired-end sequencing. A differential gene expression analysis was performed with the use of EdgeR to examine the gene expression changes with smoking cessation. A Benjamini–Hochberg corrected *P* < .05, and fold change > |1.5| was considered statistically significant. The RNA-sequencing counts were normalized with the “Voom” function in Limma R statistical software package (LogCPM) to visualize the heatmap. There were a total of 21,532 genes in the dataset after filtering for low reads in EdgeR. We explored biologic pathways associated with genes using the g:Profiler web-based tool. Gene set variation analysis (GSVA)^4^ was performed in an independent study of individuals who smoke vs those who never smoked (NORM cohort^5^) to examine whether changes on smoking cessation drive the bronchial transcriptome towards that of participants who never smoked with the use of RNA-sequencing data from bronchial biopsy specimens.^6^ This study cohort consists of 40 participants who never smoked and 37 participants who currently smoke, with 18.75 mean pack years (SD, 14.84). The group of participants who had never smoked comprised 20 male patients and 20 female patients with 104.82 mean postbronchodilator FEV_1_ percentage predicated (SD, 10.67); the participants who smoke consisted of 22 male patients and 15 female patients with 102.8 mean postbronchodilator FEV_1_ percentage predicated (SD, 9.89). GSVA formed the enrichment scores by creating an eigenvalue for a set of genes in a given sample in the context of sample population distribution. The significantly upregulated genes (false discovery rate [FDR] < .05; fold change, ≥ 1.5) that were derived from the differential expression analysis following 1 year of smoking cessation were used to create a positive enrichment score, whereas the significantly down-regulated genes (FDR < .05; fold change, ≤ −1.5) from the same analysis were used to generate the negative enrichment score. GSVA was also conducted in a second independant dataset of nasal epithelial brushings (GSE83364) of people who smoke enrolled in a 6 month smoking-cessation program.^7^ Finally, the cellular deconvolution method was applied with the use of the support vector regression approach to investigate how smoking cessation affects cellular compositions in biopsy specimens by integrating our study transcriptional profiles with previously published bronchial biopsy single-cell RNA-sequencing data^8^ with AutoGeneS,^9^ which will give the cell-specific signatures. This cellular deconvolution method will integrate the cell-specific gene expression signatures from the single-cell RNA sequencing with bulk RNA-sequencing data based on the cell-specific marker genes and provide a relative proportion of the cell-specific expression in the bulk RNA-sequencing data. The statistical analyses were done in R statistical software (version 3.5.3) with the use of the EdgeR package (version 3.24.3), GSVA (version 1.38.2), and CIBERSORT, respectively.^4^^,^^10^^,^^11^ This CIBERSORT method is an R-based package that falls under reference-based cellular deconvolution and provides a relative estimation of cell type abundance in a heterogenous bulk RNA-sequencing sample with references to cell-specific gene expression information that is obtained from single-cell RNA sequencing.

## Results

**Figure 1:**
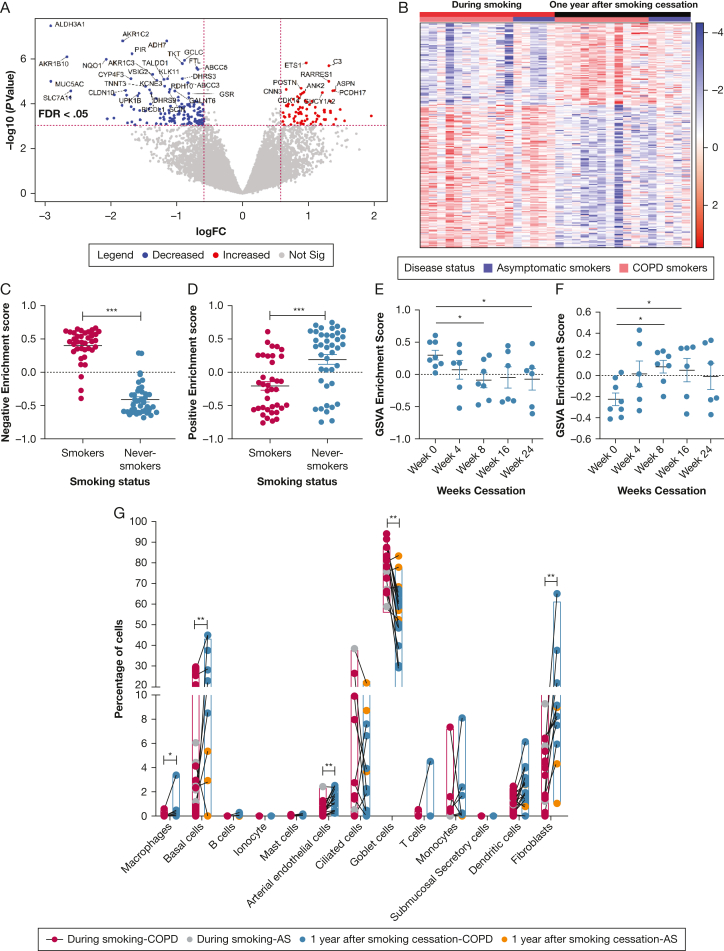
A-G, Transcriptome alterations 1 y after smoking cessation. A, Volcano plot of differentially expressed genes before and after smoking cessation. Each point represents a gene. Red and blue colors represent significantly up- and down-regulated genes, respectively. B, Heatmap shows genes significantly altered after smoke cessation. The red and blue colors represent up- and down-regulated gene-expression levels, respectively. The samples clustered based on smoking status and disease status. The red color represents smoking, black represents 1 y after smoking cessation, and pink and light blue represent COPD and asymptomatic participants who smoke. Gene set variation analysis with the RNA Seq data of participants who smoke and those who never smoked shows the same direction of (C) down-regulated (*P* < .0001) and (D) upregulated (*P* = .0001) gene expression that the participants who smoke showed 1 y after smoking cessation. ∗∗∗ = *P* value < .0001. Gene Set Variation Analysis with the RNA Seq data of nasal airway dataset (GSE83364) of participants who smoke with duration of weeks after smoking cessation shows the same pattern and significant reduction between (E) wk 0 to wk 8 (*P* = .0334) and wk 0 to wk 24 (*P* = .0470) after smoking cessation and (F) upregulated genes show the same pattern and significant increase between wk 0 to wk 8 (*P* = .0202) and wk 0 to wk 16 (*P* = .0425) after smoking cessation. ∗ = *P* < .05 (all the multiple statistical comparisons are made in one-way analysis of variance). G, Cellular deconvolution of different bronchial cell types before and after 1-y of smoking cessation: grey and yellow dots represent asymptomatic participants who smoke; red and blue dots represent participants with COPD who smoked before and after smoking cessation. The nonparametric paired (Wilcoxon) signed rank test was performed to explore the significance of these cell-type-specific signatures before and after smoking cessation. ∗ = FDR *P* value < .005, ∗∗ = FDR *P* value < .0005 AS = asymptomatic smokers; FDR = false discovery rate; GSVA = gene set variation analysis; logFC = log2 fold change.

The 11 patients with COPD and five asymptomatic participants who smoke, who were included in the present analysis had a median age of 57 years (range, 46 to 63) and 49 years (range, 45 to 57), with median pack years of 36 (range, 15 to 66) and 23 (range, 17 to 32), respectively. We found that the expression of 213 genes was significantly altered after 1 year of smoking cessation. Table 1 represents the top 25 significant genes in the differential gene expression analysis; the volcano plot in Figure 1A shows the distribution of the genes with their significance. Among these differentially expressed genes, 139 genes were lower expressed, which were enriched for xenobiotic metabolism, mainly detoxification and oxidative-stress responses (*ALDH3A1*, ADH*7*, *SLC7A11*); Aldo-Keto reductase activity (*AKR1C2*, *AKR1C3*, *AKR1B10*); mucin production (*MUC5AC*, *MUC2*); and Nrf2 pathway activity (*NQO1*, *GCLC)* like biologic pathways*.* This is in accordance with previous findings that showed that cigarette/tobacco smoke increases xenobiotic metabolism by detoxifying gas and tar phase xenobiotics in cigarette smoke.^1^ After smoking cessation, the most significantly decreased gene was *ALDH3A1*, which protects airway epithelial cells from cigarette smoke-induced DNA damage and cytotoxicity.^1^ This agrees with the highly expressed nature of *ALDH3A1* in previous studies that were conducted in the presence of cigarette smoke as a phase I xenobiotic-metabolizing enzyme.^1^ Nagaraj et al^12^ previously showed that genes that belong to the Aldo-Keto reductase family (*AKR1C1, AKR1C3*, and *AKR1B10*) are expressed highly in oral cancer cells after cigarette smoke exposure, encoding proteins involved in the detoxification of various toxic aldehydes and ketone compounds in cigarette smoke. Our analysis shows down-regulation in these Aldo-keto reductase family genes after smoking cessation, which indicates that there is no longer a need to express these genes in the airways once the toxic compounds are removed.

**Table 1:** The Top 25 Differentially Expressed Genes Altered Following Smoking Cessation

Gene Symbol	logFC	log CPM	*P* Value	FDR
*ALDH3A1*	−2.9137	9.9193	< .0001	.0005
*AKR1C2*	−1.8199	8.0983	< .0001	.0007
*ADH7*	−1.1508	8.5281	< .0001	.0007
*PIR*	−1.6790	4.9563	< .0001	.0020
*AKR1B10*	−2.6658	5.6107	< .0001	.0022
*NQO1*	−2.0697	7.2247	< .0001	.0023
*GCLC*	−0.8860	8.1401	< .0001	.0023
*ETS1*	0.9658	6.9362	< .0001	.0025
*TKT*	−0.9157	7.0533	< .0001	.0025
*C3*	1.3073	8.5922	< .0001	.0028
*FTL*	−0.6961	8.1065	< .0001	.0032
*ABCC5*	−0.6787	8.3524	< .0001	.0033
*AKR1C3*	−1.3668	6.2600	< .0001	.0052
*DHRS3*	−0.9133	6.7173	< .0001	.0066
*CYP4F3*	−1.6964	5.5313	< .0001	.0066
*KLK11*	−1.1389	5.8484	< .0001	.0066
*TALDO1*	−1.1942	6.6142	< .0001	.0066
*VSIG2*	−1.3031	5.5212	< .0001	.0068
*MUC5AC*	−2.9113	12.5110	< .0001	.0068
*RARRES1*	1.3073	5.4166	< .0001	.0068
*ABCC3*	−0.8313	6.6833	< .0001	.0075
*KCNE3*	−1.1824	5.6953	< .0001	.0101
*TNNT3*	−1.4726	4.8150	< .0001	.0101
*POSTN*	0.8871	7.0790	< .0001	.0112
*RDH10*	−1.1038	27.1830	< .0001	.0112

FDR = false discovery rate; log CPM = log counts per million; logFC = log2 fold change.

The 74 genes that were decreased after smoking cessation include genes that regulate cell-to-cell adhesion (*POSTN*, *VIM*, *PCDH17),* genes regulating cell differentiation (*CNN3, ETS1),* and apoptosis-related genes *BIRC3*. Previous studies found that serum Periostin protein levels increased in participants who previously smoked immediately after quitting smoking, which aligns with our findings.^13^ The heatmap in Figure1B represents the pattern of differentially expressed genes among each subject.

These differentially expressed genes after 1 year smoking cessation were then investigated in bulk RNA sequencing of participants who currently smoked vs those who never smoked with the use of GSVA to examine whether patterns observed on smoking are reversed toward normal by smoking cessation. Those genes lower on smoking cessation were lower in never smokers than current smokers (Fig 1C). At the same time, genes with higher expression after smoking cessation were also higher in participants who never smoked compared with those who did smoke. These findings suggest that gene expression patterns 1 year after smoking cessation reverse toward the direction of those of people who have never smoked (Fig 1D). The genes that were altered following smoking cessation were replicated in a independant nasal brushing dataset of smoke cessation over 6 months (Fig 1E, 1F).

The pathway analysis discovered that the top functional pathways associated with lower expressed genes after smoking cessation include metabolism of xenobiotics by cytochrome P450 (KEGG:00980), chemical carcinogenesis (KEGG:05204), ferroptosis (KEGG:04216), Nrf2 pathway (WP:WP2884), metapathway biotransformation phase I and II (WP:WP702), and oxidative stress (WP:WP408) (FDR, < .05). The Nrf2 pathway plays a significant role in the presence of cigarette smoke to regulate cellular protective responses to oxidative and electrophilic stress-induced damage by increasing the expression of antioxidant genes such as *NQO1*.^14^

The single-cell RNA sequencing-based cellular deconvolution findings in Figure 1G show the cellular composition shifts with smoking cessation. Among the different cell types, the percentage of goblet cells was significantly lower after quitting smoking (FDR *P* value, .0004), although basal cells (FDR *P* value, .001), fibroblasts (FDR *P* value, .0013), arterial endothelial cells (FDR *P* value, .0002), and macrophages (FDR *P* value, .004) were significantly higher after 1-year smoking cessation. We performed a subanalysis solely in patients with COPD and found similar results. Cigarette smoke exposure alters airway epithelial cell composition and induces hyperplasia of the goblet cells.^15^ The mucus hypersecretion reduction may be due to the removal of stress stimulus with smoking cessation. This removal may significantly reduce goblet cell numbers and the expression of their signature genes, such as *MUC5AC*.^15^ Conversely, the higher relative percentage of basal cells after smoking cessation may be due to active repair in the airways after smoke withdrawal^16^; however, this may also be due to the loss of goblet cell percentage that leads to the increment of other cell populations.

## Conclusion

The present study reveals the effects of 1-year smoking cessation at the transcriptomic level in airways by indicating reversible trends of oxidative stress and detoxification mechanisms. At the same time, mucus hypersecretion-associated gene expression and percentage goblet cell change that were observed in deconvolution findings further confirm that goblet cell hyperplasia and mucus production may be reversible, even to some extent in patients with COPD who smoke, with smoking cessation. Future experiments can be done to consider the reversible effects of smoking cessation to help build up COPD-related health guidelines to improve patient awareness.

## Funding/Support

This study was funded by a 10.13039/501100014780Longfonds, The Netherlands, Junior Investigator Grant 4.2.16.132JO (A. F.); S. N. H. R. is funded by the Australian government research training programme scholarship.

## Financial/Nonfinancial Disclosures

The authors have reported to *CHEST* the following: W. T. reported receiving consulting and institutional fees from Merck Sharp Dohme and Bristol-Myers-Squibb and reported conducting a role in the Dutch Society of Pathology as a board member and serving as a council member for Research and Innovation of the Federation of Medical Specialists. I. H. H. reported receiving research grants from Boehringer Ingelheim, 10.13039/100016036Health∼Holland, NOW (ZoNMW); Netherlands Scientific Organisation and received payments for working as external examiner in thesis defence Gothenborg, Sweden. ∗M. C. N. reported receiving grants from the Chan Zuckerberg Initiative, Horizon2020 program of the European Commission, and Lung Foundation Netherlands as an unrestricted research grant to the institution. ∗F. J. T. has reported receiving consulting fees from Immunai Inc., Singularity Bio B.V., CytoReason Ltd, and Omniscope Ltd, and has an ownership interest in Dermagnostix GmbH and Cellarity. None declared (S. N. H. R., B. D., B. W. M. W., W. K., B. G. G. O., M. v. d. B. and A. F.).
